# Infants prefer a trustworthy person: An early sign of social cognition in infants

**DOI:** 10.1371/journal.pone.0203541

**Published:** 2018-09-06

**Authors:** Yuiko Sakuta, So Kanazawa, Masami K. Yamaguchi

**Affiliations:** 1 Faculty of Human Life Sciences, Jissen Women’s University, Hino, Tokyo, Japan; 2 Department of Psychology, Japan Women’s University, Kawasaki, Kanagawa, Japan; 3 Department of Psychology, Chuo University, Hachioji, Tokyo, Japan; Central European University, HUNGARY

## Abstract

Recently, various studies have clarified that humans can immediately make social evaluations from facial appearance and that such judgment have an important role in several social contexts. However, the origins and early development of this skill have not been well investigated. To clarify the mechanisms for the acquisition of this skill, we examined whether 6- to 8-month-old infants show a preference for a more trustworthy-looking person. Results showed that infants preferred a trustworthy face to an untrustworthy one when both faces were high in dominance. This difference was not seen when both faces were low in dominance. Moreover, this preference disappeared when the faces were upside down. These findings suggest that the perception of trustworthiness based on facial appearance emerges in early development with little social experience. Further research is needed to verify whether infants also perceive other traits, such as competence.

## Introduction

The ability to rapidly detect a trustworthy person is important for forming good relationships within a social community. In social psychology, this ability is called “trait inference (face-to-trait inference)” or “impression judgment”.

Trait inference from a face occurs automatically, spontaneously and rapidly [1,2]. Todorov et al. have shown that it even occurs within 50 ms after exposure to a face and that trustworthiness has an effect on priming even when the face is presented subliminally [1]. Moreover, a high level of consensus in such inference among different cultures has been confirmed [3–5]. Thus, face-to-trait inference or impression judgment is a universal phenomenon. Furthermore, various studies have clarified that face-to-trait inference or impression judgment plays an important role in several social contexts, such as criminal courtroom proceedings [6,7], business [8], and political elections [9]. For example, it can affect outcomes in U.S. congressional elections [9]. Judgments about a candidate’s competency correlate strongly with judgments about that candidate’s facial attractiveness, which in turn correctly predict about 70% of congressional electoral outcomes. Thus, first impressions based on facial appearance unconsciously affect our choices or judgments in various social settings.

However, it is controversial whether appearance-based-trait inference is accurate or not. Some researchers doubt its accuracy (e.g., [10,11]) while others have shown evidence that a person’s traits can be read from his/her facial appearance to some extent (e.g., [12–14]). According to previous research, the correlation between inferred and actual traits has been verified in several studies [12,13]. Verplaetse took face pictures of target models in the prisoner’s dilemma game and showed that the participants accurately discriminated between the faces of players exhibiting cooperative and uncooperative behavior [12]. Moreover, Stirrat and Perrett [14] measured male trustworthiness and facial width, because this is a testosterone-linked trait predictive of male aggression. They found that men with greater facial width were more likely to exploit the trust of others and that other players were less likely to trust male counterparts with wide rather than narrow faces in trust games. Thus, several studies have confirmed the validity of inferring trustworthiness from facial appearance. Todorov, Funk, and Olivola [15] indicated that people can sometimes read valid information from a face, although such information is helpful only in certain contexts. That is, we should be careful about generalizing a trait inference from a face across various contexts. However, even if appearance-based-trait inference is not accurate, there is no doubt that people tend to automatically infer some traits from faces. In this paper, we focused on the perception of trustworthiness, not on actual trustworthiness.

Is it possible for young children to make impression judgments from faces? There have been few studies regarding young children’s impression judgment [16–20]. Cogsdill et al. verified that even 3-year-old children can make impression judgments in a manner similar to adults [18]. Moreover, 5- to 13-year-olds selected a captain from two people based on their perceived competence [16]. A recent study revealed that 7-month infants detected trustworthiness of faces [19], even with a subliminal presentation [20]. Thus, even young children make facial impression judgments and utilize them in social contexts similar to adults. However, this phenomenon has not been well examined at very young ages. It would be difficult for babies to make facial impression judgments if such judgments demand social experiences.

Infants obtain complex face processing skills around 5 to 8 months of age. For example, view-invariant face processing develops around 7–8 months of age [21,22] and basic recognition of facial expressions develops around 6 months of age [23]. Several studies have clarified that even infants as young as 6-months-old can differentiate between an attractive and an unattractive face, and prefer an attractive face [24,25]. Thus, it is possible that infants have a sense of social recognition in terms of facial recognition. Moreover, 6- and 10- month-old infants can perceive an individual’s social behavior (helping or hindering), and prefer an individual who helps others [26]. Therefore, we assumed that infants can make a social judgment to some extent by 6- to 8-months of age.

The current study examined whether 6- to 8-month-old infants could perceive trustworthiness from faces. We hypothesized that if such an ability requires little experience with social interaction, even infants under 1 year of age should be able to perceive trustworthiness, and would prefer a trustworthy face to an untrustworthy face. However, if social experience is necessary to perceive trustworthiness, young infants would not perceive trustworthiness and therefore would show no preference between trustworthy and untrustworthy faces.

**Fig 1:**
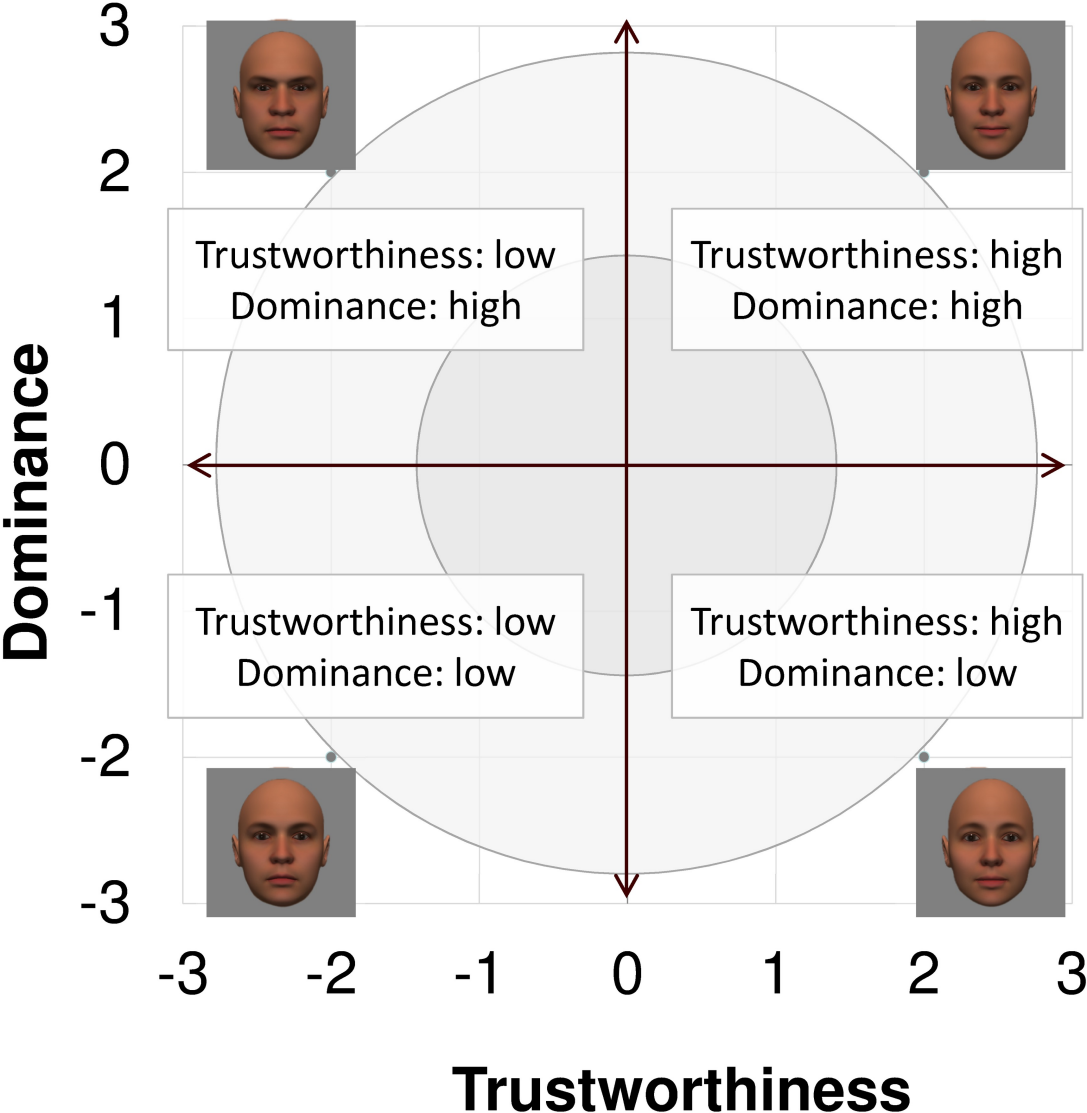
Examples of computer-generated face on a two-dimensional space of trustworthiness and dominance. This figure was made based on Oosterhof and Todorov [27]. The face images were provided from a database that was created in FaceGenModeller 3.2 (Singular Inversions, http://www.facegen.com/) and validated [27] by Prof. Alexander Todorov (Princeton University), and is open to the public (http://tlab.princeton.edu/databases/).

We used computer-generated face images [27], in which trustworthiness and dominance were manipulated, for stimuli (Fig 1). Oosterhof and Todorov [27] proposed a 2D model of face evaluation, which consists of two orthogonal axes of valence (trustworthiness) and dominance. These two axes are composite factors that have been obtained from a principal component analysis: valence evaluation includes positive judgments of attractiveness and responsibility, and dominance evaluation consists of judgments of dominance, aggressiveness, and confidence. These axes are the most important dimensions in social judgment, yet other social evaluations are also representable in this 2D model.

Kelly and colleagues have reported an other-race effect in infants [28,29]. Just like adults, infants show better discrimination for faces of their own race than ones of another race. At the same time, several studies have reported cultural universality in the trait-inference-from-faces [3–5,30], while some other studies reported both universalities and some differences between cultures [31,32]. It is possible that an other-race effect occurs for accuracy or speed of discrimination of faces, but perception of facial impressions (e.g., trustworthiness) could be more universal. Thus, we decided to use computer-generated Caucasian faces to assess the universality of perceiving facial impressions in Asian infants at this time.

Face appearance influences various social interactions. Focusing on appearance is an adaptive ability that helps us to more rapidly detect a friend or foe in a social environment. Judgment of trustworthiness can predict an individual’s social and economic success [33,34]. Todorov et al. noted that evaluating faces on valence (or trustworthiness) and dominance may be an overgeneralization of adaptive mechanisms, which attempt to estimate others’ behavioral intention and status in a hierarchy of power [35]. Our current study chose stimuli that varied on the dimensions of trustworthiness and dominance because these are important social dimensions, at least for adults. It is worth examining whether these are important for young infants as well.

We investigated whether 6- to 8-month-old infants prefer a trustworthy face in two experiments using the preferential-looking method. The faces were presented upright in Experiment 1 and upside-down in Experiment 2. When a face is inverted, face recognition performance is decreased because an inverted face prevents holistic processing [36,37]. This face inversion effect has already been confirmed in infants and even 4-month-old infants show a preference for an upright face [38]. It is possible that configuration information cannot be used for inverted faces and that facial impressions are therefore not adequately perceived [39]. If infants actually recognize facial impressions, a preference would be seen in Experiment 1, but not in Experiment 2. On the other hand, if infants just respond to some other lower-order information, their performance would be the same in both experiments.

## Experiment 1

### Method

#### Participants

Twenty-two 6- to 8-month-old infants (12 males and 10 females; mean age = 202.89 days, range = 168–251 days) participated in Experiment 1. Two additional infants were excluded from data analysis due to fussiness (1) or a side bias greater than 95% (1). All infants were full-term at birth and healthy at the time of testing. The experiments were approved by the Ethical Committee at Chuo University (2014–1). Moreover, the experiments were conducted according to the principles laid down in the Helsinki declaration. Written informed consent was obtained from each infant’s parents prior to participation in the experiment. The caregivers received a book token worth 2,000 yen for their infant’s participation.

#### Stimuli

Four computer-generated faces were chosen from a face image database [27]. Stimuli consisted of a trustworthy- and dominant-looking face, an untrustworthy-and dominant-looking face, a trustworthy- and undominant-looking face, and an untrustworthy- and undominant-looking face. These extensively validated [40] faces were created in FaceGenModeller 3.2 (Singular Inversions, http://www.facegen.com) and based on data-driven, computational models (derived from adults' judgments) of the respective traits [27,41]. All of them were created based on Caucasian faces. Using Adobe Photoshop software, the images were cropped in contour and superimposed on a uniform background of neutral grey. The stimuli are shown in Fig 1.

In the test phases, visual stimuli were presented side-by-side on a CRT monitor. The size of the pictures was 400 x 400 pixels (approximately 28 x 28 degrees of visual angles), and the distance between the images was about 214 pixels. The stimulus presentation was controlled by Python 2.5 with Vision Egg.

#### Apparatus

In the test phases, all stimuli were displayed on a Calix CDT2141A 21-inch CRT monitor (TOTOKU, Tokyo, Japan) controlled by a computer. The pixel resolution was 1024 x 768 pixels and the refresh rate was 85Hz. The infant and the CRT monitor were located inside an enclosure that was made of iron poles and covered with cloth. Each infant sat on his or her parent’s lap in front of the CRT monitor. The infant’s viewing distance was approximately 40 cm from the CRT monitor. Caregivers were asked to close their eyes during the experimental session to avoid influencing the infant’s behavior. There were two loudspeakers, one on either side of the CRT monitor. There was a CCD camera just below the monitor screen. Throughout the experiment, the infant’s behavior was videotaped through this camera. The experimenter could observe the infant’s behavior via a TV monitor connected to the CCD camera.

#### Procedure

**Fig 2:**
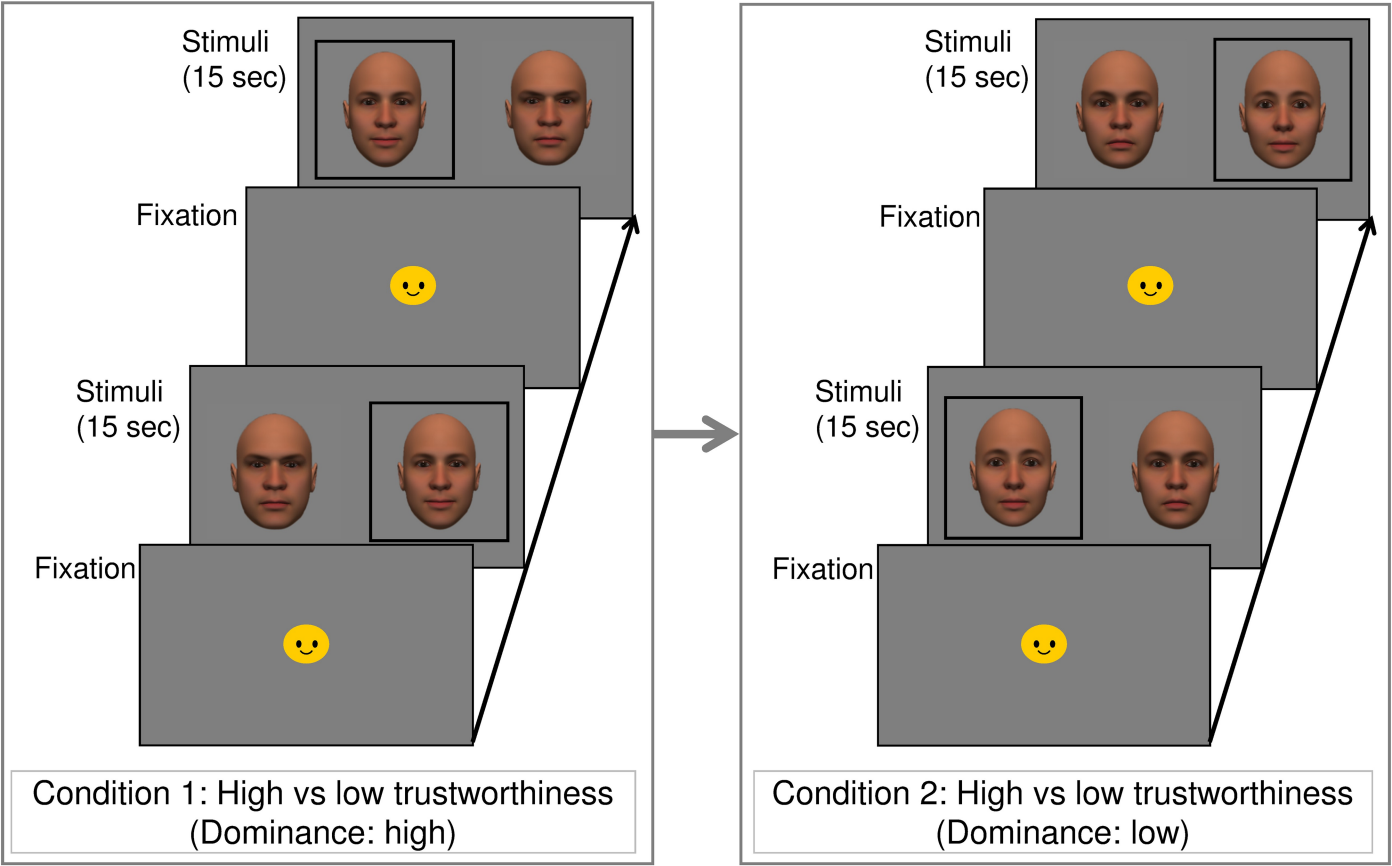
Overview of the experimental procedure. The target (trustworthy face) is enclosed with a black square. The order of conditions 1 and 2 was randomized across the participants. Some unrelated images were displayed between conditions 1 and 2. A cartoon character was presented as a fixation prior to the stimuli.

In the current study, we especially focused on trustworthiness because infants did not show a preference for high or low dominance faces in our preliminary study (n = 17). Participants viewed two pairs of trustworthy and untrustworthy faces: Both faces had high dominance in one pair and low dominance in the other pair. An overview of the experimental procedure is depicted in Fig 2.

A preferential-looking paradigm was used. Each trial began with the presentation of a colorful fixation figure (about 9.0 x 9.0 degrees of visual angle) at the center of the CRT accompanied by a short beep to attract the participant’s attention. When the infant looked at the center of the screen, the fixation disappeared and two stimuli were presented side by side. Each stimulus remained for 15 seconds. Each infant was exposed to two trials for two conditions (both were high on dominance or both were low on dominance). The faces were presented as upright. The position of the pictures was counterbalanced across the infants. A trustworthy-looking face was presented on the left in one trial and on the right in the other trial. The orders of the two trials and two conditions were randomized for each infant. Some pictures of non-face objects were presented between the two conditions to let the infants rest and maintain their interest.

#### Data analysis

Each infant’s looking time was calculated based on an offline video recorded during the experiments. In the trials, one observer, who was unaware of the stimulus identity, measured the infant’s fixations to the left and right sides of the display based on video recordings. Only the infant’s looking behavior was visible in the video. Although the observer could not see the stimulus, she could notice the timing of the beginning and the end of each trial by means of the beeping sound that was presented at those times. To compute the inter-observer agreement, a second observer’s measurement of the infants’ looking time was obtained from about 10% of the data set. Inter-observer agreement (correlation) was *r* = .87 and the reliability (Krippendorff's alpha) was .82 (calculated on the website: [42]) throughout the experiment.

The looking times were summed across the two trials for each condition. To test whether infants looked longer at one stimulus than at the other, we calculated the infants’ *preference* for the picture of the trustworthy face (target) using the following equation: [preference for target] = [looking time for trustworthy face]/[total looking time over the two test trials]*100. To examine the significance of the mean preference for the target, a two-tailed t-test versus chance (50%) was performed. In addition, we compared the preference between the faces with high and low dominance using a t test. The coefficient *r* was calculated as the effect size.

### Results and discussion

**Fig 3:**
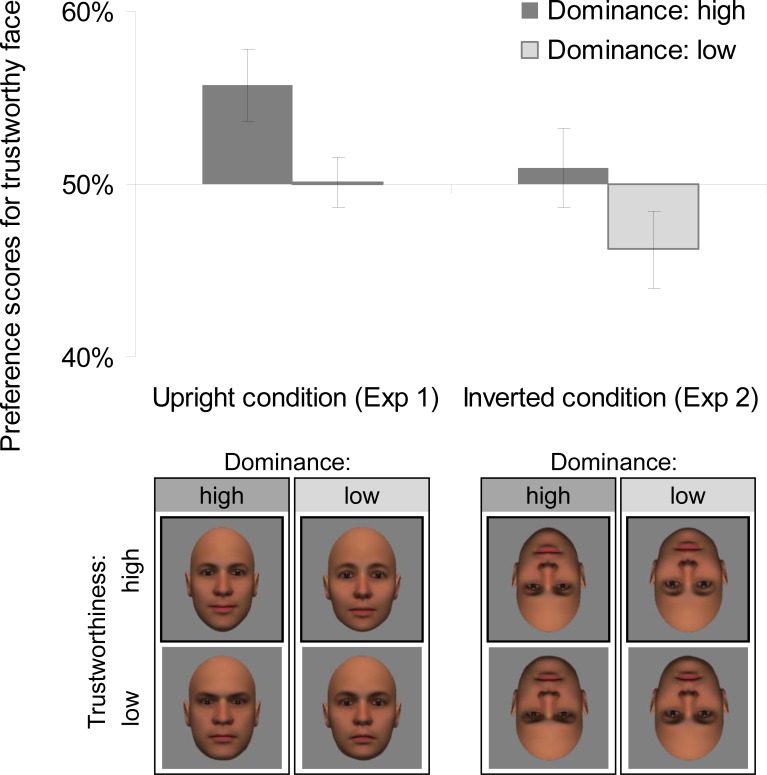
Preference scores (ratios of looking time) for trustworthy face in Experiments 1 and 2. Error bars represent Standard Error (*SE*).

The mean preference scores appear in Fig 3. To determine whether dominance differentially affected the looking time for the faces with high or low trustworthiness, we conducted a two-tailed *t*-test for the preference score with dominance as a within-participant factor. The results showed a significant difference between the preference scores for high and low dominance, *t*(21) = 2.339, *p* = .029, *r* = .46. Infants’ preference for high trustworthiness was higher for the face pair with high dominance than for the face pair with low dominance.

Next, to determine if infants preferred the trustworthy face, we conducted a two-tailed one-sample *t*-test (vs. chance level 50%) on the preference scores. This analysis revealed that infants showed a significant preference for the trustworthy face when both faces had high dominance, *t*(21) = 2.753, *p* = .012, *r* = .52. No significant difference was found when both of them had low dominance, *t*(21) = .095, *p* = .925, *r* = .02.

As for dominance, we tested the infants’ preference for high or low dominance in our preliminary study. We found no preference for either high or low dominance faces in either the high or low trustworthiness pairs (*t*(16) = .272, *p* = .789 for the high trustworthy pair; *t*(16) = .427, *p* = .675 for the low trustworthy pair). Therefore, it is thought that discriminating high and low trustworthiness is meaningful for infants, while discriminating high and low dominance is not. Taking this into account, the face with both high trustworthiness and high dominance is not always preferred. A trustworthy face is preferred only when comparing faces with high dominance. Dominance is related to hierarchy, so is important for adults who are trying to obtain social success [35]; in contrast, trustworthiness would be more important than dominance for infants. It may be suggested that the traits to be emphasized change along with one’s development.

## Experiment 2

### Method

#### Participants

Twenty-two 6- to 8-month-old infants (12 males and 10 females; mean age = 215.5 days, range = 174–252 days) participated in Experiment 2. Two additional infants were excluded from data analysis due to fussiness (1) or a side bias greater than 95% (1). All infants were full-term at birth and healthy at the time of testing. The experiments were approved by the ethical committee at Chuo University. Moreover, the experiments were conducted according to the principles laid down in the Helsinki declaration. Written informed consent was obtained from each infant’s parents prior to participation in the experiment. The caregivers received a book token worth 2,000 yen for their infant’s participation.

#### Stimuli, apparatus, procedure, and data analysis

The stimuli, apparatus, procedure, and data analysis were the same as in Experiment 1 except that the faces were presented upside-down.

### Results

The results when the faces were upside-down were not the same as in Experiment 1. There were no significant differences between the preference score and chance level for the high dominance pair, *t*(21) = .407, *p* = .688, *r* = .09 and the low dominance pair, *t*(21) = 1.673, *p* = .109, *r* = .34, and between the preference scores for high and low dominance, *t*(21) = .667, *p* = .512, *r* = .15.

## General discussion

It was revealed that 6- to 8-month-old infants differentiate faces based on the impression of trustworthiness and preferred a trustworthy face. This preference was shown when both faces had high dominance. A face with high dominance is related to physical strength and maturity whereas a face with low dominance is related to weakness and having a baby-face [27,43,44]. It is possible that a person with high trustworthiness and high dominance is preferred by infants because such a person might be a more likely protector. From an evolutionary perspective, it is highly plausible that infants would have sensitivity to a trait that would benefit them from early in their development. For example, “warmth” or “trustworthiness” would be connected to protective or nursing behavior. It would be adaptive for infants to prefer a dominant (i.e., physically strong) and trustworthy person rather than a strong and untrustworthy person because the former is more likely to protect or nurse the infant. On the other hand, a non-dominant (i.e., weak) person, whether trustworthy or untrustworthy, is unlikely to protect or nurse them. Therefore, comparing these two faces should not induce the infants’ preference. It would make sense for babies to detect and prefer such traits to help them survive.

Our results are in line with previous research [19]. It is quite surprising because even East Asian infants, who should have few chances to see Caucasian people, can perceive trustworthiness from Caucasian-looking faces. Thus, such sensitivity to trustworthiness would be culture independent. As described before, some researchers have reported the universality of trait-inference-from-faces [3,4,30]. However, there still have been few studies that examined the development of face-to-trait inferences and many issues remain to be addressed.

Our current study suggests that even young infants can make impression judgments, such as trustworthiness, based on face appearance, in an adult-like manner. Therefore, it can be thought that impression judgment does not require many social experiences as infants under 1 year of age have not experienced higher-level social interaction, such as a trust game, yet. Since the ability to detect a trustworthy person is necessary for maintaining adults’ social system, it is natural to assume that this ability requires the higher-level processing acquired through rich social experiences. However, our results suggest that young infants can obtain this ability through a slight number of social experiences after birth, such as those found in the mother-child relationship.

Interestingly, the preference for a trustworthy face was not shown when the faces were upside-down. As described in our introduction, it is plausible that configuration information cannot be used for inverted faces and that the facial impressions were therefore not adequately perceived [39]. Sakuta and Gyoba [39] have also reported that higher-level impressions, such as elegance or attractiveness, are especially decreased by inversion. Thus, social impressions are largely produced by the global configuration or proportion of a face rather than the partial features.

Our current study revealed infants’ perception of trustworthiness. However, some limitations exist because we used computer-generated male Caucasian faces in this study. Additional studies, examining factors such as gender or racial differences, will be needed to generalize our results in the future. Moreover, we only examined infants’ preference for trustworthy- or untrustworthy-looking faces in the current study. In the future, it will be necessary to examine the effect of other kinds of impressions, such as warmth, competence, and so on. Different results may be obtained because trustworthiness is thought to be especially important for infants. Studying the effects of other impressions would improve our understanding of the early development of social recognition.

In conclusion, our claim that young infants can detect trustworthiness based on facial appearance was verified. Recent studies have clarified that adults can evaluate others based on their facial appearance even when a face is presented subliminally [1]. Moreover, several studies have suggested that young children or infants can make trait inferences based on visual information [18,19], even subliminally [20]. Hence, trait inference is related to a primitive and basic disposition in humans. The current research demonstrated the possibility that, at least at 6- to 8- month of age, infants can develop sensitivity to trustworthiness in faces. This is an important issue when considering the development of human sociality.

## Supporting information

S1 File Infants’ looking time data (ms). (XLSX)
